# The Interleukin-33/ST2 Pathway Is Expressed in the Failing Human Heart and Associated with Pro-fibrotic Remodeling of the Myocardium

**DOI:** 10.1007/s12265-017-9775-8

**Published:** 2017-12-28

**Authors:** Cheyenne C. S. Tseng, Manon M. H. Huibers, Joyce van Kuik, Roel A. de Weger, Aryan Vink, Nicolaas de Jonge

**Affiliations:** 1 Department of Cardiology, University Medical Center Utrecht, Utrecht, The Netherlands; 2 Netherlands Heart Institute, Utrecht, The Netherlands; 3 Department of Pathology, University Medical Center Utrecht, Utrecht, The Netherlands; 4 Division of Heart and Lungs, Department of Cardiology, University Medical Center Utrecht, Heidelberglaan 100, Room F.01.1.46, In-house postbox E.03.511, Post office box. 85500, 3508 GA Utrecht, The Netherlands

**Keywords:** Soluble ST2, IL-33/ST2 pathway, Cardiac, Fibrosis

## Abstract

The interleukin-33 (IL-33)/suppression of tumorigenicity 2 (ST2) pathway is a potential pathophysiological mediator of cardiac fibrosis. Soluble ST2 (sST2) is one of the main isoforms of ST2 with strong prognostic value in cardiac disease. The exact role of sST2 in cardiac fibrosis is unknown. The aim of this study was (1) to investigate myocardial expression of the IL-33/ST2 pathway in relation to myocardial fibrosis in end-stage heart failure patients and (2) to study whether plasma sST2 is associated with histologically determined cardiac fibrosis. In 38 patients undergoing left ventricular assist device implantation, mRNA expression of sST2, total ST2, and IL-33 was measured in cardiac tissue obtained during the implantation. In the same tissue, histological fibrosis was digitally quantified and mRNA expression of pro-fibrotic signaling molecules, connective tissue growth factor (CTGF) and transforming growth factor beta 1 (TGFβ1), was measured. In addition, plasma levels of sST2 were determined. Expression levels of IL-33/ST2 pathway factors in myocardial tissue were significantly associated with cardiac fibrosis and the expression levels of CTGF and TGFβ1. Plasma levels of sST2 did not correlate with tissue expression of ST2, the amount of fibrosis or myocardial expression of pro-fibrotic signaling proteins. The interleukin-33/ST2 pathway is expressed in the failing human heart and its expression is associated with cardiac fibrosis and pro-fibrotic signaling proteins, suggesting a role in pro-fibrotic myocardial remodeling. Soluble ST2 levels in the circulation did not correlate with the amount of cardiac fibrosis or myocardial ST2 expression, however. Therefore, other pathophysiological processes such as inflammation might also substantially affect sST2 plasma levels.

**Electronic supplementary material:**

The online version of this article (10.1007/s12265-017-9775-8) contains supplementary material, which is available to authorized users.

## Introduction

Heart failure is a progressive disease with a large burden on public health [1, 2]. This clinical syndrome is a result of initial injury or stress followed by remodeling of the myocardium leading to changes in size, shape, and function of the heart [3, 4]. Fibrosis is an important component of ventricular remodeling and subsequent cardiac dysfunction [4, 5]. There is an urgent need for good biomarkers predicting this remodeling process. In contrast to state-of-the-art imaging techniques, biomarkers are considered mechanistically driven and clinically more useful predictors of ventricular remodeling [5]*.*

Suppression of tumorigenicity 2 (ST2) is an interleukin-1 (IL-1) receptor family member and is a transcriptional product of the IL-1 receptor like-1 (IL1RL1) gene [6]. Soluble ST2 (sST2), one of the main isoforms of ST2, is used for risk stratification in heart failure patients [7] and is considered a biomarker of myocardial fibrosis [8]. However, the exact role of sST2 and its functional ligand, interleukin-33 (IL-33), in cardiac fibrosis is unexplored [6]. The IL-33/ ST2 pathway potentially mediates myocardial inflammation and fibrosis signaling upon biomechanical strain [6, 9–11].

We recently demonstrated that end-stage heart failure patients have severely elevated sST2 levels prior to left ventricular assist device (LVAD) support. Within this group of patients, we observed a large variation that could not be explained by clinical factors such as heart failure etiology or duration and might therefore be explained by the amount of cardiac fibrosis [12]. More insight in the relation between fibrosis and ST2 may help to define strategies to predict reverse remodeling and ultimately identify LVAD patients in whom recovery of cardiac function may occur. Therefore, the aim of this study was to assess the association between cardiac fibrosis and IL-33/ST2 signaling in patients with end-stage heart failure just before LVAD implantation. The LVAD as bridge to transplantation population provides a unique opportunity to assess both plasma levels and mRNA expression in cardiac tissue in end-stage heart failure. We quantified fibrosis and pro-fibrotic signaling molecules in cardiac biopsies obtained during LVAD implantation. In addition, we assessed myocardial mRNA expression of the IL-33/ST2 pathway and measured sST2 levels in plasma.

## Method and Materials

### Myocardial Tissue and Serum

From 38 end-stage heart failure patients, myocardial tissue was obtained from the apical core biopsy during LVAD implantation. The tissue samples were split in two parts: one part was formalin-fixed and paraffin-embedded for fibrosis quantification and the other part was stored at − 80 °C for RNA isolation. Serum samples were collected within 24 h prior to LVAD implantation. All patients approved collection and banking of blood samples and tissue for research purposes and the study was approved by the institutional review board (Medisch Ethische Toetsingscommissie (METC) of the University Medical Center Utrecht, number 12/387).

### Fibrosis Quantification

The paraffin-embedded samples were sectioned at 4 μm thickness. Masson’s trichrome stain was performed with an automatic staining system (DakoCytomation Artisan, Glostrup, Denmark). Thereafter, samples were digitalized using an Aperio XT slide scanner (Aperio, Vista, CA, USA). For analysis of fibrosis, a systematic method for high-resolution digital cardiac fibrosis quantification was used as reported previously [13]. The percentage of fibrosis of the total tissue surface area was calculated with the use of Aperio Image Scope v12.1.0.5029 (Aperio, Vista, CA, USA).

### mRNA Expression

**Fig. 1:**
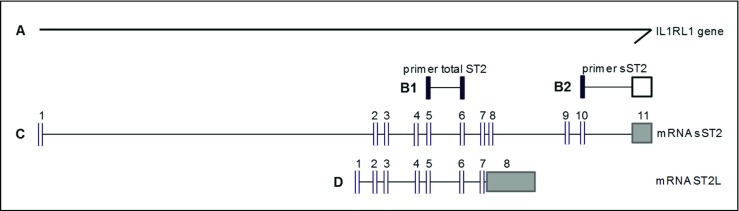
Schematic representation of the interleukin-1 receptor-like 1 (IL1RL) gene (**A**) and the intron (horizontal line)-exon (vertical stripes) structure of soluble ST2 (sST2, **C**) and ST2 ligand (ST2L, **D**) mRNA . The primer-probe combination for total ST2 (**B1**) will detect sST2 and ST2L (exon 5–6) while primer sST2 (**B2**) is specific for sST2 mRNA (exon 10–11)

RNA was isolated from 20 slides of 10 μm of frozen tissue with the RNeasy mini kit (Qiagen Inc., Austin, USA), and synthesis of cDNA was performed with superscript III, oligo-dT, and random primers (Invitrogen, Oslo, Norway). QPCR was performed as previously described on the Viia™ 7 Real-Time PCR system (Thermo Fisher Scientific, MA, USA) [14]. The mRNA expression of the IL-33/ST2 pathway and pro-fibrotic signaling proteins was analyzed. The following Taqman primer-probes were used: sST2, total ST2 (Fig. 1), IL-33, connective tissue growth factor (CTGF), transforming growth factor beta 1 (TGFβ1), and GAPDH (as housekeeping gene) (Applied Biosystems, Thermo Fisher Scientific, MA, USA). Each target was also measured in a calibrator sample (positive control). Each sample was run in duplicate. A maximum ΔCq of 0.5 between duplicates was accepted. Relative quantification (RQ) values were calculated using the comparative Cq method: relative quantity (RQ) = 2^-ΔΔCt^, ΔΔCq = ΔCq (sample) − ΔCq (calibrator), and ΔCq = Cq (housekeeping) − Cq (sample). Cq values above 35 were interpreted as negative. An estimated ratio (sST2:ST2L) was generated by calculating the percentage of ST2L, from the measured markers sST2 and total ST2 (Fig. 1). Only patients with RQ sST2 < total ST2 were included in the analysis.

### Plasma Levels of ST2

Peripheral vein blood EDTA samples were used to determine levels of sST2. After withdrawal, blood was centrifuged within 6 h. Plasma was collected in aliquots and stored at − 80 °C. The Presage ST2 ELISA assay (Critical diagnostics, San Diego, CA, USA) was used according to manufacturer’s instructions to determine sST2 concentrations in one batch [12].

### Statistics

Categorical data are expressed as number (%) and continuous data as mean ± SD or median [interquartile range (IQR), 25th–75th percentile]. Correlations were calculated using the Pearson (in case of normal distribution) or Spearman (in case of non-parametric distribution) correlation. Comparisons between two groups were performed using a Mann Whitney *U* test and between more than two groups with a Kruskal-Wallis test. Correction for multiple testing was applied when appropriate. Statistical analysis was performed with SPSS version 21 (SPSS Inc. Chicago, IL, USA). A *p* value ≤ 0.05 was considered statistically significant.

## Results

### Patient Characteristics

Thirty-eight patients were included in this study. All patients were in New York Heart Association (NYHA) class IV and received an LVAD as bridge to transplantation between January 2010 and October 2013. The majority of patients (74%) had a non-ischemic dilated cardiomyopathy (Table 1). Plasma samples and paraffin-embedded tissue of all 38 patients were available. Frozen tissue of one patient was not available for research.

**Table 1:** Patient characteristics. NYHA, New York Heart Association; CRT, cardiac resynchronization therapy; ICD, implantable cardioverter defibrillator

Characteristic	*n* = 38
Age (years)	50 (range 17–68)
Male/female	24 (63%)/14 (37%)
NYHA classification IV	38 (100%)
Heart failure duration (weeks)	220 (range 1–936)
CRT/ICD	23 (61%)
Etiology of cardiomyopathy	
Ischemic	10 (26%)
Non-ischemic	27 (71%)
Hypertrophic	1 (3%)
Inotropic medication	30 (79%)
INTERMACS profile I/II–III	10 (26%)/28 (74%)

### Myocardial Fibrosis Is Related to Expression of Pro-fibrotic Signaling Molecules

**Fig. 2:**
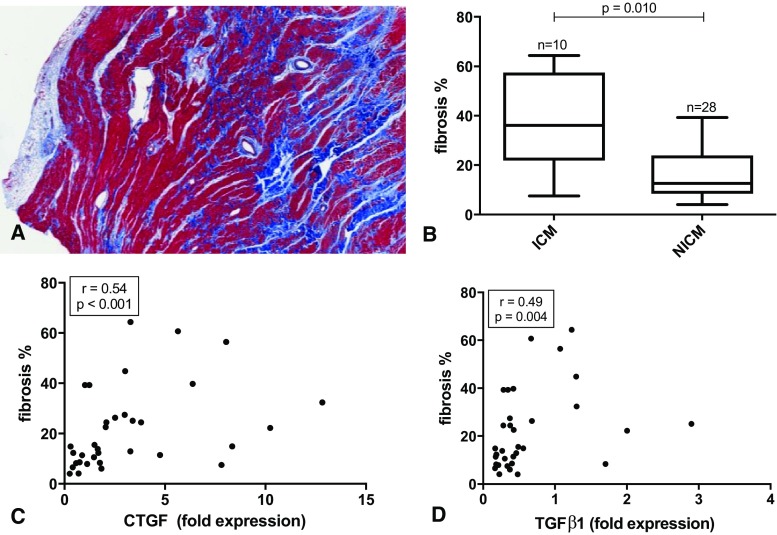
Fibrosis in heart failure and relation to pro-fibrotic proteins. **a** Digital scan of Masson trichrome stain with in red cardiomyocytes and in blue collagen deposition (fibrosis). The white area on the left is epicardial adipose tissue. **b** Histologically determined fibrosis in ischemic cardiomyopathy (ICM) vs non-ischemic cardiomyopathy (NICM). **c**, **d** Scatterplots of the correlation between the amount of fibrosis and mRNA expression of pro-fibrotic signaling molecules connective tissue growth factor (CTGF, **c**) and transforming growth factor beta 1 (TGFβ1, **d**) in myocardial tissue

The amount of fibrosis in myocardial apical tissue (*n* = 38) ranged from 4.0 to 64.4% with a median of 15.2% (IQR 10.1–28.3%). Figure 2a shows an example of the histology. Patients with ischemic cardiomyopathy had significantly more fibrosis than those with non-ischemic cardiomyopathy (*p* = 0.010, Fig. 2b). Subsequently, we studied whether the amount of fibrosis and mRNA expression of pro-fibrotic signaling molecules was related to determine whether an ongoing pro-fibrotic process was present (*n* = 37). The quantity of fibrosis was positively correlated with mRNA levels of CTGF and TGFβ1 (Fig. 2c, d; *r* = 0.54, *p* < 0.001 and *r* = 0.49, *p* = 0.004, respectively). mRNA levels of CTGF and TGFβ1 were strongly positively correlated (*r* = 0.68, *p* = 0.002) as well (supplementary Fig. S1).

### Myocardial Expression of the IL-33/ST2 Pathway Is Related to Cardiac Fibrosis

A strong positive correlation between myocardial mRNA expression (*n* = 37) of sST2 and total myocardial ST2 was observed (*r* = 0.61, *p* < 0.001, supplementary Fig. S2). The mRNA expression of IL-33 and sST2 in cardiac tissue showed a moderate positive correlation (*r* = 0.44, *p* = 0.010, supplementary Fig. S3A), while IL-33 and total ST2 demonstrated a weak non-significant correlation (*r* = 0.27, *p* = 0.121, supplementary Fig. S3B).

**Fig. 3:**
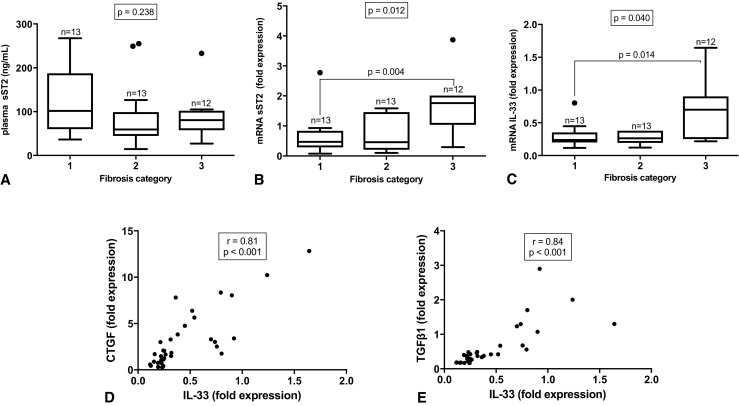
Correlation between the IL-33/ST2 pathway and fibrosis. **a** Soluble ST2 (sST2) plasma levels, **b** sST2 mRNA expression, and **c** IL-33 mRNA expression in different fibrosis categories based on tertiles. Correlation between local myocardial mRNA expression of interleukin-33 (IL-33) and pro-fibrotic markers connective tissue growth factor (CTGF, **d**) and transforming growth factor beta 1 (TGFβ1, **e**)

To determine whether cardiac fibrosis is associated with cardiac mRNA expression of the IL-33/ST2 pathway, correlations between fibrosis, pro-fibrotic signaling molecules CTGF and TGFβ1, and mRNA expression of sST2, ST2, and IL-33 were calculated (Table 2; Fig. S5). The mRNA level (*n* = 37) of sST2 was strongly positively correlated to the amount of histological fibrosis (*r* = 0.43, *p* = 0.001) and myocardial TGFβ1 mRNA (*r* = 0.54, *p* = 0.001; Fig. S5D), but not to CTGF mRNA (*r* = 0.24, *p* = 0.177; Fig. S5C). Statistically significant strong correlations were found between myocardial IL-33 mRNA levels and the amount of fibrosis (*r* = 0.46, *p* = 0.007) and pro-fibrotic signaling proteins CTGF and TGFβ1 (*r* = 0.81, *p* < 0.001 and *r* = 0.84, *p* < 0.001, respectively; Fig. 3c, d). Total ST2 mRNA was only significantly correlated with TGFβ1 mRNA expression (*r* = 0.46, *p* = 0.006; Table 2 and Fig. S5E,F).

**Table 2:** Correlations of fibrosis and the IL-33/ST2 pathway. CTGF, connective tissue growth factor; TGFβ1, transforming growth factor beta 1; sST2, soluble ST2; IL-33, interleukin-33

	Fibrosis % digital analysis		CTGF mRNA expression		TGFβ1 mRNA expression	
	*r*	*p*	*r*	*p*	*r*	*p*
sST2 plasma level	− 0.19	0.264	− 0.24	0.170	− 0.05	0.761
sST2 mRNA expression	*0.43*	*0.011*	0.24	0.180	*0.54*	*0.001*
ST2 (total) mRNA expression	0.21	0.227	0.24	0.177	*0.46*	*0.006*
IL-33 mRNA expression	*0.46*	*0.007*	*0.81*	*< 0.001*	*0.84*	*< 0.001*

Values in italics are correlations with statistical significance

Patients were then divided in categories of fibrosis based on tertiles. The amount of fibrosis from the first tertile (category 1) ranged from 4.00 to 11.42%, the second tertile (category 2) from 11.43 to 24.44%, and the third tertile (category 3) from 24.45 to 64.37%. When mRNA expression of sST2, total ST2, and IL-33 was stratified in these fibrosis categories, a significantly different expression was measured in sST2 (*p* = 0.012, Fig. 3b) and IL-33 (*p* = 0.040, Fig. 3c). A significant difference in fold expression of sST2 and IL-33 was only found between categories 1 and 3 (*p* = 0.004 and *p* = 0.014, respectively).

### sST2 in the Circulation Is Not Correlated to Myocardial ST2 Expression or Myocardial Fibrosis

As previously demonstrated, the sST2 plasma levels (*n* = 38) were strongly elevated in our cohort compared to levels in healthy individuals with a wide variation (median 74.2 ng/ml [IQR 54.7–116.9 ng/ml]; normal < 35 ng/ml) [12, 15]. To study whether these plasma levels are related to the amount of mRNA expression of total ST2 and sST2 in the myocardial tissue, matched plasma and tissue samples from the same patients were used. No significant correlation was found between sST2 plasma levels and myocardial mRNA expression (*n* = 37) of sST2 (*r* = 0.12, *p* = 0.505, supplementary Fig. S4A) or total ST2 (*r* = 0.16, *p* = 0.371, supplementary Fig. S4B). Also, no significant correlation was observed between sST2 plasma levels and the myocardial mRNA expression of IL-33 (*r* = − 0.09, *p* = 0.612, supplementary Fig. S4C) or between the ratio of sST2:ST2L (*n* = 32) and sST2 plasma levels (*r* = 0.01, *p* = 0.948, supplementary Fig. S4D).

The association between the amount of cardiac fibrosis measured with digital analysis and sST2 plasma levels was then determined (*n* = 38). No significant correlation was found (*r* = −0.19, *p* = 0.264, Table 2). Next, the correlation between sST2 in plasma and mRNA expression of pro-fibrotic signaling proteins CTGF and TGFβ1 (*n* = 37) was assessed. These factors also did not correlate significantly with sST2 levels in plasma (*r* = − 0.24, *p* = 0.170, respectively *r* = − 0.05, *p* = 0.761) (Table 2). When patients were divided in the abovementioned categories of fibrosis, plasma levels of sST2 in the three categories did not significantly differ (*p* = 0.238, Fig. 3a).

## Discussion

This study aimed to assess the association between the IL-33/ST2 pathway and histologically determined myocardial fibrosis in heart failure. The study has three important results: first, the IL-33/ST2 pathway is expressed in myocardial tissue of end-stage heart failure patients. Second, its expression is associated with local myocardial fibrosis. Third, plasma levels of sST2 did not correlate with either myocardial expression of this pathway, nor myocardial fibrosis.

We found significant correlations between the myocardial sST2 and IL-33 mRNA expression and the amount of fibrosis, suggesting a potential role of the IL-33/ST2 pathway in cardiac fibrosis. Not only the amount of fibrosis in apical tissue but also the expression of pro-fibrotic signaling molecules CTGF and TGFβ1 was significantly correlated with the IL-33/ST2 pathway factors, implying that there was an active pro-fibrotic remodeling process. Although causality cannot be concluded, these data fit the hypothesis that even in end-stage heart failure, the IL-33/ST2 pathway is involved in cardiac fibrosis and pro-fibrotic changes of the myocardium [12]. The strong correlation between the expression of IL-33 and pro-fibrotic markers was remarkable. While the sST2 mRNA expression significantly correlates with TGFβ1 and not with CTGF, and the IL-33 mRNA expression correlates strongly with TGFβ1 and CTGF, IL-33 mRNA myocardial expression might play a more important role in cardiac fibrosis than myocardial sST2 mRNA expression. Alternatively, CTGF might more specifically regulate fibrosis in the heart as the TGFβ pathway has many other functions besides modulating cardiac fibrosis and CTGF is a downstream modulator of TGFβ1 [16].

In this study with end-stage heart failure patients, sST2 plasma levels were not associated with the mRNA expression of sST2, total ST2, or IL-33 in cardiac tissue. We also found a non-significant correlation between the sST2:ST2L mRNA expression ratio and sST2 plasma levels. These results suggest that the heart may not be the main source of sST2 in peripheral blood. This observation is in line with other studies that indicate production of sST2 in. amongst others, vascular endothelial cells [17–19]. It also corresponds with the fact that various diseases are associated with high levels of sST2 in plasma [10, 20].

Since sST2 plasma levels have major prognostic value in heart failure patients and are suggested to be involved in fibrosis [8, 21, 22], an association between these two parameters seemed plausible. However, sST2 plasma levels in this study were not associated with the amount of fibrosis, nor with the expression of pro-fibrotic markers in the heart. These findings indicate that high sST2 levels in the circulation do not positively correlate with fibrosis in myocardial tissue. The lacking association of fibrosis with soluble ST2 in plasma suggests that the levels in circulation are more prominently influenced by other factors than fibrosis in the heart, e.g., chronic overload and inflammatory signals. Although a significant correlation between circulating sST2 and BNP is absent [12], inflammatory signaling due to chronic overload might cause excessive sST2 release in the circulation. This is in accordance with the significant decline in sST2 plasma levels upon LV unloading [12]. It is also in line with the concept of chronic heart failure as an inflammatory disease [23] and the role of the IL-1 family in inflammatory signaling [6]. Another explanation that sST2 in plasma and fibrosis could not be correlated in this study might be that the overall poor status of patients with end-stage heart failure (INTERMACS profile I-III) is the primary determinant of sST2 levels in plasma, as is suggested by the significantly higher sST2 levels in patients with INTERMACS profile I [12]. Perhaps, in patients with less severe decompensated heart failure and without comorbidities that interfere with ST2, plasma levels of sST2 may be more consistent with the amount of cardiac fibrosis. Furthermore, after an acute myocardial infarction, plasma sST2 levels might play a role in the process of fibrosis and remodeling [24]. In addition to patients with severe heart failure, it is also of interest to look at determinants of sST2 in the outpatient clinic where patients do not have end-stage heart failure yet, for instance, in patients undergoing a biopsy for diagnostic purposes. Besides the presented measurements pre-LVAD, it would be interesting to assess cardiac tissue post-LVAD before heart transplantation and associate fibrosis and the expression of the IL-33/ST2 pathway in this patient population. The effect of the improved neurohormonal milieu due to LV unloading may lead to diminished sST2 expression and reveal stronger associations between plasma sST2 levels and cardiac tissue expression.

The presented study emphasizes the importance of cardiac tissue analysis in the exploration of the IL-33/ST2 pathway in cardiac pathophysiology.

## Limitations

Limitations of the presented study include the end-stage heart failure population in which the development and progression of fibrosis may already be advanced to a degree that sST2 expression in the heart or in the circulation is not representative for other stages of heart failure. On the other hand, this population gives the opportunity for additional tissue analysis. Since only the apical core was assessed, this may not fully resemble the whole heart. Another drawback of this study is that inflammation was not assessed. The proposed influence of inflammatory signals on sST2 is an interesting topic for future studies.

## Conclusion

Soluble ST2 is a prognostic biomarker in heart failure patients that is suggested to be a marker of cardiac fibrosis. The results of this study show that the interleukin-33/ST2 pathway is expressed in the failing human heart and that its expression is associated with cardiac fibrosis and pro-fibrotic signaling proteins, indeed suggesting a role in pro-fibrotic myocardial remodeling. Levels of sST2 in the circulation were not correlated to cardiac fibrosis or myocardial ST2 expression. Therefore, other pathophysiological processes such as inflammation might also substantially affect sST2 plasma levels.

## Electronic Supplementary Material

ESM 1 (DOCX 1262 kb)
