# Supplementary material for: The Interleukin-33/ST2 Pathway Is Expressed in the Failing Human Heart and Associated with Pro-fibrotic Remodeling of the Myocardium
Source: J Cardiovasc Transl Res. 2017 Dec 28;11(1):15–21. doi: 10.1007/s12265-017-9775-8 (PMC5846972; doi:10.1007/s12265-017-9775-8)
Supplement: Supplementary file 1 — (DOCX 1262 kb) [file 12265_2017_9775_MOESM1_ESM.docx]

**Supplemental data**

**
Figure S1.** *Scatterplot of the correlation between cardiac mRNA expression of connective tissue growth factor (CTGF) and transforming growth factor beta 1 (TGFβ1).*

 **Figure S2.** *Scatterplot of the correlation between cardiac mRNA expression of soluble ST2 (sST2) and total ST2.*

**A**

**B**

**Figure S3.** *Scatterplots of the correlation between cardiac IL-33 mRNA expression and soluble ST2 (sST2,* ***A****) and total ST2 mRNA expression (****B****).*

**A**

**B**

**C**

**D**

**Figure S4.** *Scatterplots of the correlation between soluble ST2(sST2) levels in plasma and cardiac mRNA expression of IL-33/ST2 pathway factors. The correlation of plasma sST2 and sST2 mRNA expression (****A****), total sST2 mRNA expression (****B****), IL-33 mRNA expression (****C****) and the ratio of sST2/ST2L mRNA expression (****D****).*

**A**

**B**

**C**

**E**

**F**

**D**

**Figure S5.** *Scatterplots of the correlation between ST2 and cardiac fibrosis markers. The correlation between soluble ST2 (sST2) in plasma and myocardial expression of connective tissue growth factor (CTGF,* ***A****) and transforming growth factor beta 1 (TGFβ1,* ***B****). The correlation between myocardial expression of sST2 (****C, D****) and total ST2 (****E,F****)and CTGF and TGFβ1.*
